# Iron Metabolism in the Disorders of Heme Biosynthesis

**DOI:** 10.3390/metabo12090819

**Published:** 2022-08-31

**Authors:** Andrea Ricci, Giada Di Betto, Elisa Bergamini, Elena Buzzetti, Elena Corradini, Paolo Ventura

**Affiliations:** 1 Regional Reference Centre for Diagnosing and Management of Porphyrias, Internal Medicine Unit and Centre for Hemochromatosis and Hereditary Liver Diseases, ERN-EuroBloodNet Centre for Iron Disorders, Azienda Ospedaliero-Universitaria Policlinico di Modena, 41124 Modena, Italy; 2 Department of Medical and Surgical Science for Children and Adults, University of Modena and Reggio Emilia, 41124 Modena, Italy

**Keywords:** iron, heme, porphyria, congenital sideroblastic anemias, porphyria cutanea tarda, erythropoietic protoporphyria, congenital hereditary porphyria, acute hepatic porphyrias, X-linked sideroblastic anemia, erythropoiesis

## Abstract

Given its remarkable property to easily switch between different oxidative states, iron is essential in countless cellular functions which involve redox reactions. At the same time, uncontrolled interactions between iron and its surrounding milieu may be damaging to cells and tissues. Heme—the iron-chelated form of protoporphyrin IX—is a macrocyclic tetrapyrrole and a coordination complex for diatomic gases, accurately engineered by evolution to exploit the catalytic, oxygen-binding, and oxidoreductive properties of iron while minimizing its damaging effects on tissues. The majority of the body production of heme is ultimately incorporated into hemoglobin within mature erythrocytes; thus, regulation of heme biosynthesis by iron is central in erythropoiesis. Additionally, heme is a cofactor in several metabolic pathways, which can be modulated by iron-dependent signals as well. Impairment in some steps of the pathway of heme biosynthesis is the main pathogenetic mechanism of two groups of diseases collectively known as porphyrias and congenital sideroblastic anemias. In porphyrias, according to the specific enzyme involved, heme precursors accumulate up to the enzyme stop in disease-specific patterns and organs. Therefore, different porphyrias manifest themselves under strikingly different clinical pictures. In congenital sideroblastic anemias, instead, an altered utilization of mitochondrial iron by erythroid precursors leads to mitochondrial iron overload and an accumulation of ring sideroblasts in the bone marrow. In line with the complexity of the processes involved, the role of iron in these conditions is then multifarious. This review aims to summarise the most important lines of evidence concerning the interplay between iron and heme metabolism, as well as the clinical and experimental aspects of the role of iron in inherited conditions of altered heme biosynthesis.

## 1. Introduction

Iron (Fe) is fundamental to life in mammals. Given its remarkable property to easily switch between different oxidative states, this element is an essential cofactor for countless cellular functions which involve redox reactions. At the same time, uncontrolled interactions between iron and its surrounding milieu may be damaging to cells and tissues, for instance, through the production of reactive oxygen species (ROS) [1].

Heme—the iron-chelated form of protoporphyrin IX—is a complex, macrocyclic molecule by which our body manages to exploit the catalytic, gas-binding, and oxidoreductive properties of iron in the most diverse biochemical settings. In fact, most of the iron reserves in mammalian cells are routed to the pathway of heme biosynthesis—the remainder being employed in other prosthetic groups, i.e., iron–sulfur [Fe-S] clusters. The majority of the body production of heme, in turn, is incorporated into hemoglobin, the major oxygen-carrying protein in mammals—thus, regulation of heme biosynthesis by iron is central in erythropoiesis. Additionally, heme is a cofactor in several essential metabolic pathways (i.e., nitric oxide biosynthesis and signal transduction, tryptophan, and homocysteine metabolism), which can be modulated by iron-dependent signals as well.

Two disease groups, collectively known as porphyrias and congenital sideroblastic anemias, are both caused by an impairment in some steps of heme biosynthesis. In porphyrias, according to the specific enzyme involved, heme precursors accumulate, up to the enzyme stop, in disease-specific patterns and organs [2]. Therefore, these diseases manifest themselves under strikingly different clinical pictures [2]. In sideroblastic anemias, several distinct dysfunctions in different enzymes—more or less strictly involved with heme biosynthesis—cause an abnormal utilization of mitochondrial iron by erythroid precursors [3].

In line with the complexity of the processes involved, the role of iron in porphyrias and congenital sideroblastic anemias is multifarious [4,5,6]. This review aims at summarising the most important lines of evidence concerning the interplay between iron and heme metabolism, as well as the clinical and experimental aspects of the role of iron in inherited conditions of altered heme biosynthesis.

## 2. Role of Iron in the Biosynthesis of Heme

**Figure 1:**
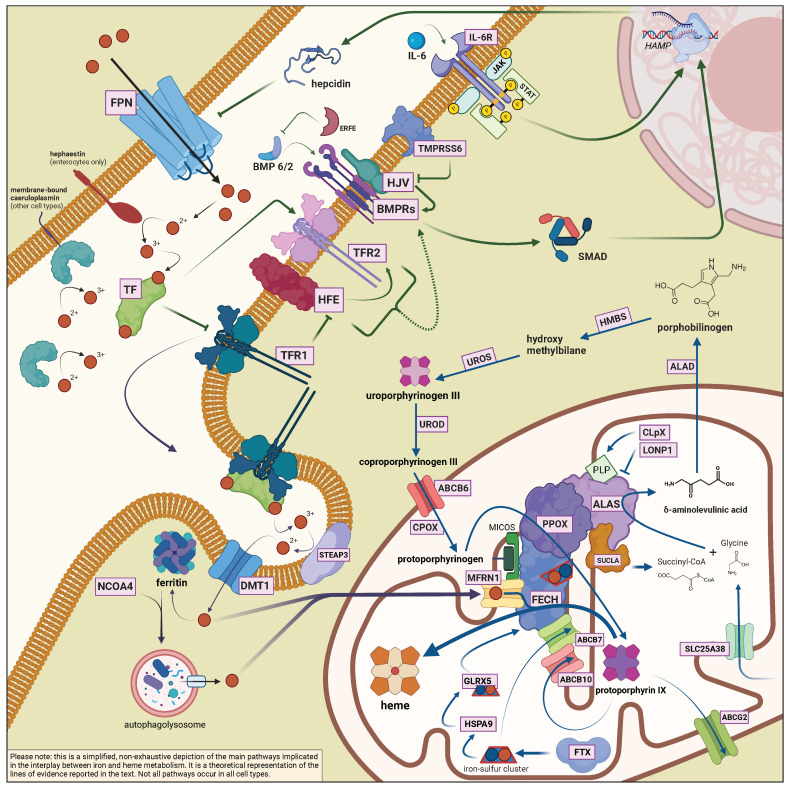
**From iron to heme.** Both iron metabolism and heme biosynthesis are complexly regulated to efficiently exploit the properties of iron in the most diverse biochemical settings. The mechanisms and factors depicted are described in detail in Section 2. ALAD, δ-aminolaevulinate dehydratase; ALAS, δ-aminolaevulinate-synthase; ABCB6, ATP-binding cassette super-family B member 6; ABCB7, ATP-binding cassette super-family B member 7; ABCB10, ATP-binding cassette super-family B member 10; ABCG2, ATP-binding cassette superfamily G member 2; BMP 6/2, bone morphogenetic protein 6 and 2 dimer; BMPRs, bone morphogenetic protein receptors; CLpX, caseinolytic mitochondrial matrix peptidase chaperone subunit X; CPOX, coproporphyrinogen III oxidase; DMT1, divalent metal transporter 1; ERFE, erythroferrone; FECH, ferrochelatase; FPN, ferroportin; FTX, frataxin; GLRX5, glutaredoxin 5; *HAMP*, hepcidin gene; HFE, human homeostatic iron regulator protein; HJV, hemojuvelin; HMBS, hydroxymethylbilane synthase; HSPA9, heat-shock protein family A member 9; IL-6, interleukin 6; IL-6R, interleukin 6 receptor; JAK, Janus kinase; LONP1, lon peptidase 1, mitochondrial; MICOS, mitochondrial contact site and cristae organizing system; MFRN1, mitoferrin 1; NCOA4, nuclear receptor coactivator 4; PLP, pyridoxal phosphate; PPOX, protoporphyrinogen oxidase; SLC25A38, mitochondrial solute carrier family member 25 A38 (glycine transporter); SMAD, small mother against decapentaplegic (protein family); STAT, signal transducer and activator of transcription protein; STEAP3, the six-transmembrane epithelial antigen of prostate 3 (metalloreductase); SUCLA, succinyl-CoA synthase; TF, transferrin; TFR1, transferrin receptor 1; TFR2, transferrin receptor 2; TMPRSS6, matriptase; UROD, uroporphyrinogen III decarboxylase; UROS, uroporphyrinogen III synthase. The red circles represent iron atoms; when needed, ferrous (^2+^) or ferric (^3+^) states are indicated. Created with Biorender.com (last accessed on 25 August 2022).

The main actors of iron and heme metabolism, which are mentioned in this section, are summarized in Table 1 and Table 2, with a non-exhaustive list of the known associated diseases. Figure 1 recapitulates the main metabolic pathways in the interplay between iron and heme biosynthesis (not all pathways occur in all cell types).

### 2.1. General Aspects of Iron Metabolism in Mammals

Under physiologic conditions, the total content of iron in the human body is about 3–4 g—less in females than males. While iron entry and mobilization are tightly regulated by several proteins, iron excretion is possible only through the turnover of the intestinal epithelium, skin cell or hair shedding, menstrual cycles, or blood losses. Crucially, the recycling of heme-bound iron represents one of the main sources for maintaining adequate supplies (together with mobilization of iron reserves from the liver), whereas the intestinal absorption of dietary iron, under physiologic conditions, only compensates for daily losses. In macrophages, heme recycling is realized through heme oxygenase (HO), which extracts iron in its ferrous form—releasing carbon monoxide in the process—and decomposes the protoporphyrin ring into carbon dioxide and biliverdin IXa—the latter is eventually reduced to bilirubin. With regards to dietary intakes, instead, non-heme iron is a substrate of divalent metal transporter 1 (DMT1) after being reduced to its ferrous form (Fe^2+^) by duodenal cytochrome B (DCYTB) at the apical membrane of duodenal and proximal jejunal enterocytes [7].

Whether from reticuloendothelial cells or enterocytes, iron is exported into the bloodstream by ferroportin (FPN1, coded by the gene *SLC40A1*)—the only known iron exporter [8]. This is a crucial step in the entire cycle of iron homeostasis, and the only which can be endogenously tuned in the exchange of iron with the environment: in fact, by regulating iron release from enterocytes and the reticuloendothelial system, ferroportin ultimately determines iron circulating levels. Ferroportin membrane expression can be chiefly modulated through vesicle internalization: in particular, hepcidin (HAMP) triggers ferroportin polyubiquitination by direct binding and thereby leads to its degradation in lysosomes [9]. More recently, it has been reported that hepcidin can occlude the central cavity of ferroportin, thus blocking iron export also by means of an endocytosis-independent mechanism [10].

To adequately regulate systemic iron homeostasis in physiological processes, hepatic HAMP expression is influenced by several factors, such as plasma and tissue iron levels, various erythropoietic stimuli (e.g., hypoxia or anemia), and infections or other causes of inflammation [11,12]. At the intracellular level, HAMP expression is chiefly regulated by the bone morphogenetic protein (BMP)/suppressor of mothers against the decapentaplegic (SMAD) signal transduction pathway [13,14].

It has been proposed that when tissue iron stores increase, liver sinusoidal endothelial cells (LSECs) enhance the expression of BMP6 and BMP2, which are thereby secreted and likely form heterodimers [15]. On the hepatocyte cell membrane, BMP6 and BMP2, most likely in the form of heterodimers, bind to a tetramer composed of BMP type I (activin receptor-like kinase—ALK-2 and 3) and type II (activin receptor—ACTR- 2A and BMP receptor 2) receptors and complexed with the co-receptor hemojuvelin (HJV). As a result, the intracellular signal transductors SMAD1, SMAD5, and SMAD8 are phosphorylated and bind SMAD4 to enter the nucleus and trigger HAMP expression. Transmembrane protease serine 6 (TMPRSS6), instead, has a negative effect on HAMP expression: when tissue iron levels decrease, TMPRSS6 cleaves HJV, decreasing hepcidin levels and rebalancing the iron stores [11,12,15]. When plasma iron levels increase, iron-loaded transferrin binds transferrin receptor 1 (TFR1) and transferrin receptor 2 (TFR2) on the hepatocyte membrane, inducing an increase in TFR2 protein stability and likely weakening the interaction between TFR1 and the human homeostatic iron regulator protein (HFE) [15]. As a consequence, HFE dissociates from TFR1 and forms a complex with TFR2 to start a signal transduction cascade to the *HAMP* gene, possibly interacting with HJV and the BMP/SMAD pathway and ultimately increasing hepcidin levels [11,12].

Moreover, hypoxia and anemia stimulate erythropoiesis and act as negative regulators of HAMP expression. In hypoxic or anemic conditions, the kidney produces higher amounts of erythropoietin (EPO), which in turn promotes erythroferrone (ERFE) production by red blood cell precursors. Then, ERFE binds BMPs, interfering with BMP-BMP receptor interaction and the following signaling cascade, thus resulting in decreased hepcidin levels [11,12]. Both infections and inflammation result in the secretion of interleukin 6 (IL-6) by macrophages. IL-6 induces the activation of the Janus kinase (JAK)-signal transducer and activator of transcription 3 (STAT3) signaling pathway in hepatocytes, leading to the upregulation of HAMP expression [11,12].

In the bloodstream, iron is taken up by TF after being oxidized to its ferric form (Fe^3+^) by membrane-bound hephaestin (HEPH) or the copper-carrying enzyme ceruloplasmin (CP). Transferrin is capable of carrying two atoms of ferric iron, which can be released to cells through endocytosis by binding to TFR1; during this process, iron is eventually reduced to its ferrous form by the metalloreductase six-transmembrane epithelial antigen of prostate 3 (STEAP3) and transported through DMT1. At the intracellular level, up to 4500 ferrous iron atoms can be stored in ferritin, which is composed of 24 subunits of light (FTL) and heavy chains (FTH1), differently combined. Iron uptake is dependent on FTL, whereas FTH1 displays ferroxidase properties [16]. The stored iron is then released and made available to the cell through lysosomal degradation of ferritin (ferritinophagy), in a process mediated by nuclear receptor coactivator 4 (NCOA4) [17].

Iron delivery to mitochondria could be accomplished through either a ferritin-dependent or a ferritin-independent pathway: in the former, a role in regulating the iron flux has been reported for NCOA4 and poly rC–binding protein 1 (PCBP1), a cytosolic iron chaperone which mediates iron delivery to ferritin [18]; in the ferritin-independent pathway, iron could be directly transferred to the mitochondrion from the outside through transferrin-containing endosomes [19,20]. Both pathways seem to play a role in erythropoiesis and heme biosynthesis [18,19,20].

Concerning the regulation of several proteins involved in iron homeostasis, a very versatile, post-transcriptional mechanism is represented by the interaction between iron regulatory proteins (IRP1 and IRP2) and the iron response element (IRE) in the 5${}^{\prime }$- or 3${}^{\prime }$-untranslated regions (UTRs) of mRNAs [21]. In conditions of iron deprivation, IRPs bind avidly to IREs. When intracellular iron is high, instead, IRP1 incorporates a [4Fe-4S] cubane cluster and gains aconitase activity, releasing the IRE, whereas IRP2 undergoes ubiquitination and proteosomal degradation. IREs, in turn, are highly conserved RNA stem-loops and differentially regulate the translation of their transcript according to where they are located. For instance, in the condition of iron deficiency, ferritin and ferroportin are negatively regulated by an IRP-IRE interaction in the 5${}^{\prime }$-UTR, while TFR1 and DMT1 translation is enhanced by IRP binding to the IREs in their 3${}^{\prime }$-UTRs. As explained in the following Section (see Section 3.3), this regulatory mechanism may be bypassed in the final steps of erythropoiesis to allow for the highly increased demand for iron required for hemoglobinization [22].

Among the several known disorders of iron metabolism, hemochromatosis (HC) is the most common inherited cause of primary iron overload [23]. A homozygous pathogenic variant (p.C282Y/p.C282Y) in *HFE* is the cause of the classic form of HC [24,25], whereas other rarer forms of HC are caused by pathogenic variants in other genes involved in iron metabolism, namely *TFR2*, *HAMP* or *HJV*; additionally, uncommon gain-of-function mutations in *SLC40A1* which cause ferroportin to be resistant to hepcidin activity are associated to a phenotype indistinguishable from HC. Loss-of-function variants in *SLC40A1*, instead, give rise to a distinct form of iron overload (ferroportin disease). Furthermore, several other inherited conditions are associated with alterations in the metabolism of iron and iron overloads, such as iron deficiency–iron refractory anemia (IRIDA, caused by pathogenic variants in *TMPRSS6*), aceruloplasminemia or hypoceruloplasminemia, and iron-loading anemias [3,24,26,27].

### 2.2. The Interplay between Heme Biosynthesis and Iron

From a molecular standpoint, heme, as iron protoporphyrin-IX, is a cyclic tetrapyrrole and a coordination complex for diatomic gases (e.g., oxygen, carbon monoxide, nitric oxide), accurately engineered by evolution to exploit the redox properties of iron while minimizing its damaging effects on tissues. When incorporated into hemoglobin—or myoglobin—heme serves the irreplaceable function of the oxygen carrier, but this molecule is also fundamental, as a prosthetic group, to the correct functioning of several other hemeprotein, such as cytochromes, catalases, nitric oxide synthases, soluble guanylate cyclases or enzymes involved in the metabolism of some amino acids (tryptophan/serotonin, methionine/homocysteine, and others) [28]. In general, every cell in the body needs heme to carry out some vital metabolic processes.

At the same time, an excess of free heme can be toxic to tissues: several mechanisms of damage have been described [29,30], many of which culminate with the release of ROS through iron-catalyzed Fenton reactions. It should also be remarked that heme, as a lipophilic molecule, intercalates into lipid membranes, either reacting within the hydrophobic phospholipid bilayer and yielding lipoperoxides, or quickly entering cells, thus precipitating cytolysis or causing intracellular damage to proteins or DNA [29,30]. From a clinical standpoint, heme toxicity becomes a threatening issue in all those conditions which are burdened by hemolysis, such as sickle cell disease, β-thalassemia, malaria, ischemia-reperfusion, hemolytic-uremic syndrome, paroxysmal nocturnal hemoglobinuria or even severe sepsis [31], as well as rhabdomyolysis with myoglobinuria. As a detoxifying countermeasure, free heme can be efficiently scavenged by hemopexin, an acute phase plasma glycoprotein, and secondarily albumin [32]. The kidney is particularly susceptible to heme toxicity; in this regard, fundamental protection against heme-induced tubular damage is provided by the inducible form of heme oxygenase (HO-1), whose expression is enhanced, among others, by free heme itself, cytokines, and several oxidant species [33,34]. Anecdotally, a transient decrease in kidney function has been reported in a patient suffering from acute intermittent porphyria after therapy with heme arginate was vigorously implemented to treat a porphyric attack [35]. Other than the inducible HO-1, two other HO isoenzymes have been identified: in particular, HO-2 is a constitutive isoform that plays a role in the modulation of signaling to tissues through the synthesis of carbon monoxide, a diatomic gas with vasodilatory and neurotransmitting properties similar to nitric oxide [36,37].

The major heme-synthesizing organ in humans is, understandably, the bone marrow, which accounts for ∼80% of total heme production and requires around 20 mg of iron supply daily [22]; the liver, in turn, produces ∼15% of total heme in the body, followed, in terms of overall contribution, by the kidney [38,39].

Heme biosynthesis is realized through an eight-step metabolic pathway [28,40]: in animals, it starts with the rate-limiting enzyme aminolevulinate synthase (ALAS), which produces δ-aminolevulinic acid (ALA) from glycine and succinyl-CoA, in the so-called Shemin pathway. Two isoforms of ALAS exists: ALAS1 is constitutively expressed by every cell in the body, whereas ALAS2 is specific to erythroid tissues and is expressed at ∼30 fold higher levels than its counterpart in the liver [2]. Notably, the *ALAS2* gene is on the X chromosome (Xp11.21). Both forms need pyridoxal phosphate (PLP) as a cofactor.

Consistent with their different evolutionary purposes, the two isoforms are regulated by different stimuli: ALAS1 is under a negative feedback by heme, whereas ALAS2 is repressed by iron deficiency. The translation of ALAS2 depends on iron availability through the presence of an IRE in the gene 5${}^{\prime }$-untranslated region (5${}^{\prime }$-UTR): in a setting of iron deficiency, IRPs bind to the IRE, inhibiting ALAS2 translation [41,42], even though this mechanism may be by-passed in the terminal steps of erythropoiesis (see Section 3.3) [22]. Recently, a new mechanism of post-translational regulation of ALAS1 has been proposed in human cells, involving the ATP-dependent proteases lon peptidase 1, mitochondrial (LONP1), and caseinolytic mitochondrial matrix peptidase chaperone subunit X (ClpX): both proteases seem to be involved in the negative feedback regulation of heme biosynthesis through heme-dependent degradation of ALAS1 [43,44]. Additionally, a significant increase in ALA levels has been observed in mouse erythroleukemia cells knock-out for the *ClpX* gene, together with decreased PPOX and FECH activities, resulting in anemia and PPIX accumulation—a phenotype reminiscent of erythroid protoporphyria. The authors of this study suggested that CLPX could also regulate ALAS2 turnover, increasing its proteolytic degradation, and concomitantly optimize PPOX and FECH activities with molecular mechanisms which still remain unknown [45]. Intriguingly, ClpX also promotes ALAS activity by catalyzing the insertion of PLP [46]. Thus, opposite regulatory mechanisms operate within the same molecular machinery, possibly allowing a better fine-tuning of the enzyme activity.

In the second step of heme biosynthesis, two molecules of ALA are condensed by ALA dehydratase (ALAD) to form porphobilinogen (PBG), which undergoes polymerization into hydroxymethylbilane (HMB) by HMB synthase (HMBS). Afterward, HMB undergoes cyclization by uroporphyrinogen III synthase (UROD) to yield uroporphyrinogen III, which is further modified to coproporphyrinogen III by uroporphyrinogen III decarboxylase (UROD), then to protoporphyrinogen by coproporphyrinogen oxidase (CPOX) and protoporphyrin IX by protoporphyrinogen oxidase (PPOX). Finally, in the last step of heme biosynthesis, an atom of ferrous iron is chelated into the protoporphyrin IX ring by the enzyme ferrochelatase (FECH). The first and last three steps of the pathway happen in the mitochondrion, the middle ones in the cytoplasm—in fact, coproporphyrinogen III is transported through the mitochondrial transported ABCB6, whereas the exact mechanisms underlying ALA translocation are not entirely clear. Noteworthy, ALAD, HMBS, and uroporphyrinogen III synthase (UROS) have both a “house-keeping” and an erythroid-specific promoter in their transcript, although only HMBS displays two different isoforms –erythroid and non-erythroid [2].

Ferrochelatase, a homodimeric enzyme located on the inner surface of the inner mitochondrial matrix [47], represents a fundamental crossroad between porphyrin and iron metabolism [48]. Notably, FECH activity is enhanced through a signaling pathway that culminates with FECH phosphorylation and is activated by erythropoietin (EPO) [49].

FECH possesses a [Fe-S] cluster, which confers structural stability to the enzyme [50] and possibly endows it with redox sensing functions [51]. Apparently, FECH receives iron from mitoferrin, a mitochondrial solute carrier that is essential for the proper assembling of heme [52]. Additionally, a role in the delivery of iron to FECH could be played by frataxin [53], which binds to FECH [54] but also contributes to the assembling of [Fe-S] clusters [55]. It also appears that the topological integrity of the mitochondrial cristae is essential to the correct functioning of FECH [56]. Even though FECH can accept several divalent metal ions as substrates, it is deemed that in vivo, only iron and zinc have access to it through mitochondrial transporters. Interestingly, some divalent heavy metals (e.g., Hg and Pb) can be accepted as a substrate, but the product of the chelation is poorly released from the enzyme [57].

Intriguingly, it has been reported that mice lacking one or two functional copies of the mitoferrin 1 (*Mfrn1*) gene in hepatocytes, when fed ALA, show an increase in protoporphyrin IX production, with histological liver alterations (chronic cholestasis, early-to-advanced fibrosis) reminiscent of erythropoietic protoporphyria (EPP) [58]. The authors of the study suggested that the observed protoporphyrin IX build-up could be chiefly caused by an insufficient iron supply to the mitochondria—due to a decreased/absent function of mitoferrin 1, which could not be replaced by mitoferrin 2. In mice not treated with ALA, alterations in Mfrn1 were not associated with signs of protoporphyria, possibly because of the Irp1-mediated suppression of Alas2—in fact, Mfrn1-deficient mice start to develop protoporphyrin accumulation when the interaction between Alas2 and Irp1 is impaired [59]. Furthermore, it has been shown that MFRN1 mRNA levels strongly correlate with FECH activity, both in conditions of normal and impaired FECH expression [60].

In the last few decades, many different proteins have been involved in the assembly and transport of [Fe-S] clusters: adenosine triphosphate binding cassette subfamily B member 7 (ABCB7) is the main mitochondrial [Fe-S] exporter to the cytosol; heat-shock protein family A member 9 (HSPA9) is a mitochondrial chaperone essential to the transfer of newly formed [2Fe-2S] clusters to glutaredoxin 5 (GLRX5); GLRX5, in turn, represents the immediate donor of [2Fe-2S] clusters to target apoproteins, such as FECH, respiratory complex proteins, or IRP1 cytosolic aconitase. Defects in the production or activity of each of these proteins are responsible for an impairment in the functioning of [Fe-S] clusters, which causes an increased iron uptake and reduced iron utilization, ultimately leading to the clinical picture of sideroblastic anemias (see Section 4.1.1 and Section 4.2.2) [61].

It has been recently reported that at least ferrochelatase, protoporphyrinogen oxidase, and aminolevulinic acid synthase-2 form a mitochondrial complex (metabolon) [62], which may possibly include additional factors involved in the transport of iron (mitoferrin) and porphyrin (ABCB10), [Fe-S] cluster biogenesis (ABCB7), and ALAS2 substrate synthesis (succinyl-CoA synthetase) [62,63,64,65]. Topological proximity is certainly convenient for all these factors which handle iron within the mitochondrial matrix in that it may constitute a safeguard mechanism against the excessive spillover of such potentially toxic element in a milieu very sensitive to oxidative damage.

**Table 1:** Main actors of iron metabolism in mammals.

Protein	Gene	Function	Associated Diseases
Bone morphogenetic protein 2	*BMP2*	Ligand of the BMP-SMAD signaling pathway regulating hepcidin expression in response to iron	
Bone morphogenetic protein 6	*BMP6*	Ligand of the BMP-SMAD signaling pathway regulating hepcidin expression in response to iron	BMP6-associated iron-overload
Caeruloplasmin	*CP*	Soluble/membrane-bound copper-carrying ferroxidase	Aceruloplasminemia
Divalent metal transporter 1	*DMT1* or *SLC11A2*	Ferrous iron importer	DMT1 deficiency
Duodenal cytochrome B	*DCYTB*	Reduces dietary ferric iron to ferrous form at the apical border of enterocytes	
Erythroferrone	*ERFE*	Hepcidin inhibitor, produced by the bone marrow in response to erythropoietin	
Ferritin heavy chain	*FTH1*	Subunit of ferritin, with ferroxidase activity	FTH-related iron-overload
Ferritin light chain	*FTL*	Subunit of ferritin, with iron storage properties	Hyperferritinemia-cataract syndrome Hereditary benign hyperferritinemia Neuroferritinopathy L-ferritin deficiency [66]
Ferroportin	*SLC40A1*	Ferrous iron exporter	Ferroportin disease (loss-of-function)*SLC40A1*-related hemochromatosis (gain-of-function)
Frataxin	*FXN*	Iron carrier, participates in iron-sulfur cluster biogenesis	Friedreich’s ataxia
Haephastin	*HEPH*	Membrane-bound ferroxidase	
Hemojuvelin	*HJV* or *HFE2*	BMP co-receptor, involved in the iron-sensing pathway which regulates hepcidin	*HJV*-related hemochromatosis
Hepcidin	*HAMP*	Iron regulating hormone; internalises ferroportin	*HAMP*-related hemochromatosis
Human homeostatic iron regulator protein	*HFE*	Protein involved in the iron-sensing pathway which regulates hepcidin	*HFE*-related HH
IRE binding protein 1	*IREB1* or *IRP1* or *ACO1*	Iron-sensing regulator of translation; aconitase activity in the presence of iron	
IRE binding protein 2	*IREB2* or *IRP2*	Iron-sensing regulator of translation	*IRP2*-related protoporphyria
Matriptase	*TMPRSS6*	Cleaves membrane-bound HJV; negative regulator of hepcidin in response to iron deficiency	Iron-deficient iron refractory anaemia (IRIDA)
Mitoferrin-1	*MFRN1*	Intramitochondrial iron carrier	
Nuclear receptor coactivator 4	*NCOA4*	Delivers ferritin to autophagolysosomes (ferritinophagy)	
Six-transmembrane epithelial antigen of prostate 3	*STEAP3*	Membrane-bound metalloreductase	Sideroblastic anaemia with primary hypogonadism
Transferrin	*TF*	Ferric iron carrier	Hypo/Atransferrinemia
Transferrin receptor	*TFRC*	Receptor for endocytosis-mediated iron uptake; one of the plasma iron sensors	TFRC-related combined immunodeficiency [67]
Transferrin receptor 2	*TFR2*	Protein involved in the iron-sensing pathway which regulates hepcidin	*TFR2*-related hemochromatosis

**Table 2:** Main actors of heme metabolism in mammals.

Protein	Gene	Function	Associated Diseases
δ-aminolaevulinate dehydratase	*ALAD*	Dehydrates ALA to yield PBG	ALAD-deficiency (Doss) porphyria
δ-aminolaevulinate synthase 1	*ALAS1*	Condenses glycine and succinyl-CoA to yield ALA	
δ-aminolaevulinate synthase 2	*ALAS2*	Condenses glycine and succinyl-CoA to yield ALA (erythroid-specific isoform)	X-linked congenital sideroblastic anaemia (loss-of-function) X-linked erythropoietic protoporphyria (gain-of-function)
ATP-binding cassette super-family B member 6	*ABCB6*	Imports porphyrins into mithocondria	Phenotype modifier in porphyrias [68]
ATP-binding cassette super-family B member 7	*ABCB7*	Mitochondrial [Fe-S] cluster exporter	X-linked sideroblastic anaemia with ataxia
ATP-binding cassette super-family B member 10	*ABCB10*	Mitochondrial exporter with putative roles in porphyrin or iron transport; complexes with MFRN1 and FECH to enhance heme biosynthesis	
ATP-binding cassette super-family G member 2	*ABCG2*	Cytosolic and mitochondrial exporter of protoporphyrin IX; also involved in the export of heme	
caseinolytic mitochondrial matrix peptidase chaperone subunit X	*CLpX*	Mitochondrial protein with ATP-dependent protease and unfoldase activity; regulates ALAS turnover; activates ALAS catalyzing PLP insertion	Phenotype modifier in protoporphyria (see Section 3.3)
coproporphyrinogen III oxidase	*CPOX*	Eliminates two carboxyl groups from coproporphyrinogen III side chains to yield protoporphyrinogen IX	Hereditary coproporphyria (autosomal dominant) Harderoporphyria (autosomal recessive)
ferrochelatase	*FECH*	Chelates iron into protoporphyrin IX to yield heme	Erythropoietic protoporphyria
glutaredoxin 5	*GLRX5*	Mitochondrial protein with thiol reductase activity; involved in [Fe-S] cluster assembly	Autosomal recessive sideroblastic anaemia
hydroxymethylbilane synthase	*HMBS*	Condensates four PBG molecules into HMB	Acute intermittent porphyria (AIP)Autosomal recessive AIP
heme oxygenase 1	*HO-1*	Cleaves heme into biliverdin IXα, releasing CO and ferrous iron (inducible isoform)	HO-1 deficiency
heme oxygenase 2	*HO-2*	Cleaves heme into biliverdin IXα, releasing CO and ferrous iron (constitutive isoform); involved in the CO signalling pathway	
hemopexin	*HPX*	Heme scavenger in the plasma	
heat shock protein family A member 9	*HSPA9*	Mitochondrial protein with chaperone activity for [Fe-S] clusters	Autosomal recessive sideroblastic anaemia
lon peptidase 1, mitochondrial	*LONP1*	ATP-dependent protease involved in the turnover of mitochondrial matrix protein	CODAS (Cerebral, Ocular, Dental, Auricular and Skeletal) syndrome [69]
protoporphyrinogen oxidase	*PPOX*	Dehydrogenates protoporphyrinogen IX to yield protoporphyrin IX	Variegate porphyria (VP) Autosomal recessive VP
mitochondrial solute carrier family 25 member A38	*SLC25A38*	Mitochondrial glycine transporter	Autosomal recessive sideroblastic anaemia
succinyl-CoA synthase	*SUCLA*	Controls the flux of heme precursors catalyzing a reversible conversion from succinate + coenzime A to succynil-CoA (precursor of ALA)	
uroporphyrinogen III decarboxylase	*UROD*	Eliminates four carboxyl groups from uroporphyrinogen III side chains to yield coproporphyrinogen III	Porphyria cutanea tarda (sporadic or familial)Hepatoerythropoietic porphyria
uroporphyrinogen III synthase	*UROS*	Converts linear PBG to cyclic uroporphyrinogen III	Congenital erythropoietic porphyria (Günther disease)

## 3. Clinical and Experimental Aspects of the Role of Iron in Porphyrias

### 3.1. Porphyria Cutanea Tarda

Porphyria Cutanea Tarda (PCT) exists both as an inherited disorder (familial PCT, F-PCT), caused by a heterozygous mutation of uroporphyrinogen III decarboxylase (UROD), and as an acquired entity (sporadic PCT, S-PCT) [70]. More than two-thirds of patients with PCT belong to the latter group, and even those with a pathogenic variant of UROD do not usually develop F-PCT unless in the presence of other factors [71]. Namely, PCT is strongly associated with conditions of iron overload [72,73,74,75], such as hemochromatosis, hepatitis C virus infection, and alcoholism. PCT is an almost exclusively cutaneous disorder, presenting with sunlight-sensitive, painful, blistering lesions (bullae, erosions) which heal slowly and leave scars, most typically in the back of hands or around the mouth; hyperpigmentation or hypertrichosis can occur as well.

Patients with PCT almost universally benefit from iron depletion through phlebotomy, whereas iron supplementation therapy unleashes or exacerbates the symptoms [75,76,77]. Beneficial effects were also noticed after implementing some dietary changes, i.e., with a vegetable-fruit, low-calorie (∼500 Kcal/day) diet, which would reduce the total iron content of the body and improve symptoms and biochemical signs of PCT [78].

Compared to non-porphyric controls, significant differences have been found in PCT patients regarding the presence of variants associated with HFE-related HC [74,79,80,81,82,83,84]. One study found that ∼20% of PCT patients were homozygous for the pathogenic variant p.C282Y∖p.C282Y in the *HFE* gene (the typical genotype associated with the risk of developing HFE-HC [25]), ∼7% were compound heterozygous p.C282Y∖p.H63D (which can predispose, in the presence of other acquired factors, to a phenotype reminiscent of an “intermediate”, milder form of HC). Consistent with this, the frequency of heterozygous p.C282Y, p.H63D, or homozygous p.H63D∖p.H63D genotypes (which are not usually associated per se to pathologic iron accumulation) were not significantly different between PCT patients and controls [74]. All patients with PCT had clear biochemical or histological signs of iron overload, with homozygous p.C282Y∖p.C282Y patients with PCT displaying the worst phenotype. Additionally, the allelic frequency of the p.H63D variant was significantly higher in chromosomes from F-PCT compared to S-PCT patients [74].

Interestingly, a useful animal model for studying PCT is represented by *Urod${}^{+\setminus -}$, Hfe${}^{-\setminus -}$* mice, which are also carriers of a homozygous null mutation in *Hfe*, other than a heterozygous null mutation in *Urod* [85].

Even though hepatic UROD is produced at half the normal levels in F-PCT patients, both forms of PCT present a reduction in hepatic UROD activity to ∼25% [86,87,88], leading to suspect the presence of an inhibiting compound. In fact, it has been demonstrated that uroporphyrinogen undergoes iron-dependent partial oxidation of one bridge carbon of the tetrapyrrolic macrocycle to yield uroporphomethene, a competitive inhibitor of UROD [88], in a reaction likely catalyzed by cytochrome 1A2 of the P450 family (CYP1A2) [88,89].

### 3.2. Congenital Erythropoietic Porphyria

Congenital erythropoietic porphyria (CEP)—also known as Günther’s disease—arises from a biallelic impairment of uroporphyrinogen III synthase (UROS)—or, less frequently, X-linked hematopoietic transcription factor GATA-binding factor 1 (GATA1) [90,91]. In CEP, UROS deficiency reroutes the metabolic pathway of hydroxymethylbilane towards non-enzymatic cyclisation, which yields porphyrin isomer I metabolites - uroporphyrinogen I and coproporphyrinogen I. The latter cannot be further processed by coproporphyrinogen III oxidase -CPOX, an enzyme stereospecific for the III isomer- and both metabolites are non-enzymatically oxidized to uroporphyrin I and coproporphyrin I, respectively [91,92].

In CEP, the severity of the clinical manifestations varies widely, ranging from mild photosensitivity, with cutaneous symptoms reminiscent of PCT, to devastating blistering lesions leading to deformities by photomutilation (i.e., loss of fingers, eyelids, ears, as well as other sunlight-exposed areas). Anemia is present as the result of both intravascular hemolysis, which can be very severe, and ineffective erythropoiesis. Of note, a few cases have been described of patients with hematological disorders in whom the replication of clonal myeloid subsets with somatic mutations resulted in acquired phenotypes similar to CEP [93,94]. Liver involvement is unusual, although hepatopathy caused by fibrosis and signs of diffuse intrasinusoidal extramedullary hematopoiesis (characterized by the presence of erythroid precursors within markedly congested and dilated sinusoids), possibly a consequence of moderate to severe chronic anemia, has been described [95]. In patients with CEP, bone marrow transplantation is potentially curative and should be considered, in the presence of a suitable donor, according to the phenotype, genotype, and biochemical profile of the disease [96].

Until recently, most of the available therapeutic options were based on a pioneering report of a patient whose symptoms of CEP could be satisfactorily managed with hypertransfusion therapy (blood hematocrit above 39%), aiming at nearly complete suppression of endogenous erythropoiesis, coupled with iron chelation by deferoxamine to manage transfusion-related iron overload [97]. In this patient, the urinary iron excretion was maximal and the urinary porphyrin content was minimal after 6 days since blood transfusion (under deferoxamine): this lag would account for the time required for the bone marrow to adapt and suppress endogenous erythropoiesis after transfusions [97]. Similar to patients with transfusion-dependent thalassemias, liver and heart iron overload with high levels of serum iron and ferritin can be burdensome complications of chronic hypertransfusion, notwithstanding iron chelation therapy [96]. Furthermore, at least one case has been described concerning a pediatric CEP patient with transfusion-related kidney iron overload and nephrotic syndrome [98].

Quite recently, it has been first reported the case of a CEP patient who underwent a remarkable improvement in her symptoms of porphyria in the setting of iron depletion caused by gastrointestinal bleeding [95]. Following this anecdotal finding, the patient started an off-label iron-chelating therapy with deferasirox, which effectively reduced uroporphyrin and coproporphyrin urinary levels and alleviated the disease phenotype [95]. In vitro studies on the bone marrow from the patient (or her sibling) showed that partial iron restriction, obtained by an intermediate ratio of holo- to apo-transferrin in the culture medium, was the most effective in allowing the maturation of UROS-deficient erythroid cells [95]. Furthermore, a decrease in ALAS2 activity and porphyrin levels could be observed, in the same cell population, after iron deprivation in vitro [95].

These findings have proved instrumental in disclosing the possibility of inducing iron deficiency in patients with CEP to alleviate the disease phenotype. Given the higher risk of adverse events associated with iron chelation, iterative phlebotomies have been proposed as a safer means to achieve iron depletion -understandably, this approach is only feasible in patients without severe anemia [6,99,100]. To avoid an abrupt compensatory induction of erythropoiesis, gradual increases in the volume of blood withdrawn during phlebotomies has sometimes been attempted, starting from small quantities [99].

Concerning the physiopathology of the disease, interesting results have been disclosed in vivo by comparing the phenotype of iron overload, with signs of severe hemolytic anaemia, in a CEP mouse model [101] with models of hemojuvelin (*Hjv${}^{-\setminus -}$*)-related hemochromatosis [102]. While *Hjv${}^{-\setminus -}$* mice displayed severe iron overload in hepatocytes, with no iron deposits in the spleen, in animals with CEP the iron accumulation was observed predominantly in Kupffer cells, with spleen enlargement as a manifestation of compensatory extramedullary erythropoiesis, and high levels of total iron content in the spleen. Although high levels of ferritin could be measured in both models, both serum iron and transferrin were elevated in CEP mice, with normal transferrin saturation, whereas *Hjv${}^{-\setminus -}$* mice showed high transferrin saturation. Importantly, CEP mice displayed early signs of iron deficiency in the course of erythropoietic differentiation, such as low reticulocyte hemoglobin content, as well as signs of enhanced compensatory erythropoiesis, such as an increase in erythropoietin levels and bone morphogenic protein 4, and suppression of hepcidin. Notably, in CEP models, ferroportin was increased in enterocytes and liver, and an accumulation of iron in the kidney cortex, most pronounced in proximal tubules, was highlighted by Perl’s staining. In sum, an efficient erythroid response to intravascular hemolysis could be observed in this particular CEP mouse strain, albeit a following study of the same group showed that different patterns of iron overload and alterations of iron metabolism can be observed in different CEP mouse strains, according to their specific pathogenic variant [103].

It has been suggested that *ALAS2* expression, which is dependent on iron status through the IRP/IRE system, can act as a disease modifier in CEP [104,105], thus suggesting a rationale for therapeutic iron depletion. Interestingly, delivery of short hairpin RNAs through lentiviral vectors has been attempted to silence ALAS2 or HMBS in *UROS*-deficient CD34+ cells derived from a CEP patient: this RNA interference approach yielded a significant reduction in porphyrin overload and disclosed a correlation between porphyrin accumulation and ALAS2 expression [106]. Similar results were also obtained with the oral iron chelator deferiprone, which could also reverse the skin symptoms, hematological abnormalities, and liver/spleen lesions (decrease in erythroid clusters from extramedullary erythropoiesis) in CEP mouse models [106].

### 3.3. Erythropoietic and X-Linked Protoporphyria

In the final step of heme biosynthesis, iron is complexed into a protoporphyrin IX ring by the enzyme ferrochelatase (FECH) [107].

Patients with erythropoietic protoporphyria (EPP) carry an inactive copy of the *FECH* gene in one allele, together with an *in trans* intronic single-nucleotide variant (c.315-48T>C, previously known as IVS3-48T/C), which corresponds to a common hypomorphic haplotype [108]. Therefore, EPP displays an autosomal pseudo dominant pattern of inheritance, with patients being usually compound heterozygous even though homozygous genotypes have been described [108,109]. Patients with EPP have a FECH activity which is ∼10–30% of the normal [108]. A gain-of-function mutation of ALAS2, instead, gives rise to X-linked protoporphyria (XLP), a rarer condition which has been discovered only in 2008 and presents with almost identical clinical findings. In both EPP and XLP, protoporphyrins accumulate in multiple sites, most notably the bone marrow, the liver, and the skin. Consistent with this, patients with EPP and XLP present with mild microcytic hypochromic anaemia and thrombocytopenia, which were found to be strongly correlated with higher erythrocyte protoporphyrin IX content [110]; liver disease, which is caused by accumulation in the biliary system of hydrophobic protoporphyrins may precipitate a life-threatening acute cholestatic hepatitis in a small percentage of cases and cutaneous photosensitivity symptoms, perhaps the most burdensome for patients, may suffer from a range of painful, burning skin reactions when exposed to sunlight or artificial light of a particular wavelength (e.g., emitted by surgical lamps which are not filtered on purpose) [107,110,111,112,113].

Several modifier genes have been identified, which may contribute to the severity of the phenotype of protoporphyrias: in particular, an abnormal transcript of mitoferrin-1 has been identified in patients with a known mutation leading to EPP or XLP, as well as some patients with a phenotype consistent with protoporphyria but no other identified mutation [114].

Differently from other conditions, such as lead intoxication, primary iron deficiency, or other red blood cells disorders, the protoporphyrins which accumulate are metal-free, rather than zinc-chelated; in XLP, zinc-protoporphyrins usually exceed 15% of the total, whereas in EPP they are less than 15%. This implies that the levels of zinc-protoporphyrins cannot be fully relied upon to grade the severity of iron deficiency in these patients.

Both EPP and XLP patients often present with signs of iron deficiency, such as low serum ferritin levels (often lower than 20 ng/mL) which may be refractory to iron supplementation [111,112,115,116,117]. An early study estimated that EPP patients could not have more than 250 mg of iron stores which could be readily mobilized after phlebotomy [72]. Several studies reported a strong association between serum ferritin and hemoglobin levels, together with the absence of significant signs of chronic disease anemia [110,116,118]. At least in humans, ringed sideroblasts and mitochondrial iron deposition have been reported [119].

Interestingly, it has been highlighted that soluble transferrin receptor and serum iron levels are often unaltered in patients with EPP [110,115,116,118], which led some authors to conclude that iron deficiency would not be a strong limiting factor in the synthesis of hemoglobin and erythropoiesis [110]. Noteworthy, it has been shown that hepcidin levels in EPP patients are decreased as an appropriate measure to iron deficiency [115,116], and gastrointestinal absorption of ferrous sulfate is normal [115], which would exclude an impairment in the intestinal absorption of iron [115]. In this regard, a mouse model of EPP failed to show any improvement in the biochemical and histological signs of the disease after being treated with an RNA interference agent targeting TMPRSS6 to mimic a condition of IRIDA, in which hepcidin is up-regulated [120].

Intriguingly, it has been hypothesized that, at least in FECH-deficient mice, a compensatory enhancement of extramedullary erythropoiesis causes a redistribution of iron reserves from peripheral tissues to the spleen while maintaining intact the total iron content of the body [121].

It has also been shown that serum transferrin levels positively correlate with erythrocyte protoporphyrin IX content [110], which would corroborate the hypothesis, first proposed in FECH-deficient mice [121], that protoporphyrin IX may induce the hepatic synthesis of transferrin, functioning as a sensor of intracellular iron shortage to the erythroid lineage. Increased protoporphyrin IX levels have also been significantly associated with male gender and growth/differentiation factor 15 (GDF15) levels [116].

Of note, phlebotomy has been attempted in patients with EPP and severe liver damage, in an approach similar to CEP, with promising results in terms of reduction in serum protoporphyrin levels and markers of liver injury: understandably, these findings were paralleled by a decrease in hemoglobin and serum ferritin [122].

The effects of iron supplementation therapy in EPP and XLP are controversial [4,5,6]. Early reports mention a beneficial effect of iron supplementation on EPP symptoms, as well as protoporphyrin and transaminase levels [123,124,125], whereas other authors warned against a worsening of cutaneous and hepatic symptoms [126,127,128]. Some beneficial effects of iron supplementation have been observed in patients with XLP [117] and it has been claimed that the clinical and biochemical improvement previously reported in patients with EPP was due to them having XLP instead [117,129]. In the latter condition, iron supplementation would enhance the activity of a normally functioning FECH, coupling with the substrate excess of protoporphyrin IX and converting it to heme [117,129]. On the other hand, benefits from iron supplementation in EPP patients could also derive from chelation of protoporphyrins in the guts in the context of their enterohepatic circulation, non enzymatic intracellular chelation, or iron-induced enhancement of FECH activity [117,125].

From a biochemical perspective, iron would enhance the activity of both ALAS2—through the IRP/IRE regulatory system—and FECH—which requires iron both as a substrate and for its [Fe-S] clusters. Therefore, iron supplementation may be beneficial or detrimental according to the ratio of activation between the two ends of the heme biosynthetic pathway. In human erythroleukemic cells, it has been demonstrated that iron chelation increases the mRNA levels of ALAS2, but not FECH, while decreasing the protein levels of both. Thus it has been suggested that ALAS2 transcription may be induced—through as-yet-unknown mechanisms—in conditions of impaired FECH activity, but ALAS2 translation would still be stopped by means of the IRP/IRE interaction. In EPP, iron depletion would then result in a safeguard mechanism, as it would impede ALAS2 overexpression with a subsequent increase in protoporphyrin IX levels [130].

Interestingly, it has been reported that in the terminal steps of erythroid maturation, the IRP/IRE regulatory mechanisms may be bypassed to allow high expression of both ALAS2 and TFR1 while maintaining low ferritin levels [22]. In this study, the authors used primary mouse erythroid cells, which allowed them to closely follow the terminal steps of erythroid differentiation, including full hemoglobinization and enucleation [22]. In particular, a difference could be noticed between self-renewing erythroid progenitors, which maintain the precursor cell pool and are under the classic iron-dependent IRP/IRE regulation, and maturing erythroblast, which instead show an uncoupling between ALAS2, ferritin, and TFR1 expression. From a physiologic perspective, this would permit us to reach the saturating levels of iron needed for massive hemoglobinization in the terminal stages of erythropoiesis while not wasting resources in iron storage, which would be futile at this stage [22]. It may be speculated whether an exacerbation of such mechanisms may intervene in the context of EPP, in which very low ferritin levels may be an expression of enhanced, but poorly effective, erythropoiesis. In fact, it has been reported an enhanced expression of ALAS2 in young erythrocytes and higher levels of ALAS2 mRNA in the peripheral blood cells of EPP patients, which would be unexpected given their putative status of iron deficiency [129]. Moreover, FECH inhibition through N-methyl-protoporphyrin led to an increase in ALAS2 mRNA in an in vitro model of erythroleukemic cells [129], a finding which was also reported in mice fed with griseofulvin (which is converted to N-methyl-protoporphyrin in the liver) [131]. As mentioned in Section 2.2, a putative uncoupling mechanism could involve CLPX-dependent regulation of ALAS2 [132]. In this regard, a clinical and biochemical phenotype consistent with protoporphyria (including sideropenic anemia) has been recently reported in a female patient who harbored two different mutations: one in the IRE of *ALAS2*, inherited from the maternal lineage (whose members were asymptomatic), and another in the *CLPX* gene, which was shared with the patient’s father and uncle (who presented both with high protoporphyrin levels, but only mild photosensitivity) [133,134]. Functional studies on this particular mutation in *CLPX* suggested that the mutated protein may form heterohexamers with the wild-type and act as a dominant-negative to impair ALAS turnover, thus increasing protoporphyrin IX levels as well as ALAS protein levels and activity [133].

#### IRP2-Related Protoporphyria

It is worth mentioning that mice which lack IRP2 expression (IRP2${}^{-\setminus -}$) display a peculiar phenotype, featuring Alas2 overexpression, markedly elevated levels of protoporphyrin IX (metal-free > zinc), and an increase in levels of *Hmbs* mRNA in the spleen, with also skin and eye lesions [135]. Additionally, IRP2${}^{-\setminus -}$ mice show microcytic anaemia with severe iron depletion in the bone marrow and markedly reduced levels of transferrin receptor in erythroid precursors, albeit with hyperferritinemia and percentages of transferrin saturation comparable to wild-type controls. Several of these manifestations are more severe when also one IRP1 allele is non-functional (IRP1${}^{-\setminus +}$ IRP2${}^{-\setminus -}$) [135]. The most notable symptom in IRP2-deficient mice, though, is adult-onset neurodegeneration, likely caused by an abnormal regulation of iron metabolism, with axonal degeneration, neuronal loss, and signs of iron overload in axonal structures and oligodendrocytes [136,137]. Intriguingly, the total content of iron in the brain and spleen is unchanged relative to controls, whereas the total content of iron in the liver is increased [135].

In 2019, the first case of a human with bi-allelic loss of *IREB2* (coding for IRP2) was described, recapitulating the phenotype observed in animals: the patient was a 16-year old with neurodegeneration, microcytic hypochromic anemia refractory to iron supplementation, mildly elevated ferritin levels with normal serum transferrin and iron levels, low transferrin receptor expression, and a marked increase in zinc-protoporphyrin IX levels. Strikingly, the clinical picture could be reversed by lentiviral gene therapy, which restored IRP2 expression. No photosensitivity has been reported in this patient [138].

### 3.4. Acute Hepatic Porphyrias

Acute hepatic porphyrias (AHPs) are caused by heterozygous pathogenic variants of *HMBS* (acute intermittent porphyria—AIP– which is the most common), *CPOX* (hereditary coproporphyria—HCP), *PPOX* (variegate porphyria—VP), or, in the case of the exceedingly rare ALAD-deficiency porphyria, by pathogenic variants of *ALAD* in homozygosis [139]. Of note, lead intoxication acts through the steric hindrance of ALAD, thus recapitulating the corresponding inherited condition [140].

Patients with AHPs are at risk of acute neurovisceral attacks (acute porphyric attacks—APAs), which can pose a serious threat in terms of survival or long-term impairment. APAs can be unleashed by exogenous or endogenous stimuli that supposedly increase the metabolic demand for heme, thus enhancing ALAS expression. As a consequence, given the enzymatic deficiency down the biosynthetic pathway, heme precursor levels increase manifold. Consistent with the enzymes involved, in AIP only ALA and PBG accumulate, whereas in HCP and VP there is also an increase in cyclic porphyrin levels, which gives rise to phototoxic cutaneous manifestations reminiscent of PCT. Patients with AHPs are also burdened by long-term complications, such as arterial hypertension, chronic neuropathy, porphyria-associated kidney disease, or non-cirrhotic hepatocellular carcinoma, which can be further debilitating [141].

The build-up of non-porphyrin heme precursors (ALA and PBG) is deemed to be a major mechanism of damage in AHPs [140], albeit relative heme deficiency may also play a role [142]. In the presence of iron and oxygen, ALA undergoes a phosphate-catalyzed auto-enolization to yield the oxidizing species superoxide anion (O_2_·), HO· radical, and ALA enoyl radical (ALA·) [143,144]. In the following step, ALA· is oxidized by iron to 4,5-dioxovaleric acid (DOVA), a DNA alkylating agent [145] to which guanine moieties are particularly susceptible in the presence of ferritin [146]. Crucially, it has been shown that the ALA enoyl radical causes ferritin to release Fe^2+^ ions [144,147]. In fact, ALA-driven oxidation may selectively damage the tryptophan and cysteine moieties of apoferritin, thus altering its secondary and tertiary structure and impairing FTL-dependent iron uptake (but not FTH1-dependent ferroxidase activity) [148].

Additionally, in the brain of AIP animal models (either rats treated with succinyl acetone methyl ester or intraperitoneally injected with ALA), increases in total non-heme iron, ferritin, and lipid peroxidation have been reported, together with enhanced antioxidant activity of the copper-zinc superoxide dismutase [149,150]. Higher non-heme iron levels have also been observed in the liver of AIP animal models [147].

The oxidative damage exerted by ALA may have more general effects on iron homeostasis as well as incubation with ALA increases IRP1 activity: in this regard, a cell-permeable antioxidant as N-acetylcysteine was able to revert this condition, in contrast with extracellular-acting catalases or superoxide dismutases [151].

Additionally, ALAS1 activity is enhanced by fasting through the key action of a transcriptional coactivator, peroxisome proliferator-activated receptor-gamma coactivator 1 (PGC-1α) [152]; at the same time, signalling through PGC-1α enhances hepcidin expression during starvation, with subsequent ferroportin degradation, low serum iron levels, and iron accumulation in the liver [153].

In AHPs, the negative feedback exerted by heme on the expression of ALAS1 also has a clinical significance: acute porphyric attacks can be managed with heme arginate (hemin) infusions, which downregulate ALAS1 and reduce circulating ALA levels. Until recently, off-label prophylactic hemin infusions have been the only therapeutic option for patients with several or markedly severe APAs. As a foreseeable complication of this approach, patients under hemin maintenance therapy are prone to develop (liver) iron overload with high serum ferritin levels.

In recent years, a novel siRNA-based agent, givosiran, has been approved for the treatment of AHPs [154]. Givosiran exploits the RNA-induced silencing complex (RISC) machinery to inhibit ALAS1 mRNA translation. Treatment with givosiran has been very effective in reducing the annualized rate of APAs and improving the overall quality of life of patients with AHPs [154,155,156], although some adverse effect must be taken into consideration and caution should be warranted as to the occurrence of unexpected others. Of interest, at least some adverse events may be possibly related to drug-induced relative heme deficiency in its non-hematopoietic routes of utilization: for instance, an impairment in heme biosynthesis induced by givosiran may play a role in the observed alterations of homocysteine catabolism [157,158,159,160,161,162,163], cytochrome P450 function [164], or nitric oxide synthase-regulated kidney microcirculation [141,165].

## 4. Clinical and Experimental Aspects of the Role of Iron in Congenital Sideroblastic Anaemias

Congenital sideroblastic anemias (CSAs) are a group of rare anemias characterized by both an altered use of mitochondrial iron by erythroid precursors and an accumulation of ring sideroblasts in the bone marrow [3].

CSAs are caused by pathogenic variants in several genes involved in the biosynthesis of heme (*ALAS2*, *SLC25A38*, and *FECH*), [Fe-S] cluster assembly (*ABCB7*, *GLRX5*, *HSPA9*, *HSBC*), as well as the translation of other mitochondrial proteins (*TRNT1*, *PUS1*, *YARS2*, *SLC19A2*, *NDUFB11*). The latter group is mainly responsible for syndromic sideroblastic anemias, which are usually associated with milder anemia without systemic iron overload, and whose description goes beyond the aim of this review [166,167]. Instead, an overview of CSAs caused by defects of [Fe-S] cluster assembly is provided, as increasing lines of evidence have supported the direct role of [Fe-S] cluster biogenesis in the heme biosynthetic pathway, e.g., by affecting the supply of succinyl-CoA substrate, the IRP-mediated regulation of ALAS2 translation, and the post-translational regulation of FECH activity [50,168].

The most common form of CSA is *ALAS2*-related, X-linked sideroblastic anemia (40% percent of all CSA cases), followed by autosomal recessive sideroblastic anemias (ARCSA), which account for 35–40% of cases. Pathogenic variants in *SLC25A38* cause <10% of all CSA cases [169].

Mitochondrial iron overload occurs in all sideroblastic anemias: in sideroblasts (which are erythroblasts containing iron inclusions) iron deposits in perinuclear mitochondria and is visible as a “ring” when cells are stained with Perl’s blue. Ringed sideroblasts may also be present in other conditions characterised by alterations in iron metabolism, such as myeloproliferative disorders or copper deficiency [170].

It has been demonstrated that in sideroblastic anemias, most of the iron in excess is contained in mitochondrial ferritin (MtF)—a mechanism deemed to protect organelles from oxidative damage [171,172]. Even though the translation of cytosolic ferritin is regulated by means of an IRP1/IRE interaction, a recognizable IRE has not been identified in the gene for Mtf. In analogy with frataxin down-regulation in Friedreich ataxia, feedback inhibition of the MtF gene (*FTMT*) by protoporphyrin IX synthesis has been proposed [173].

A common pathophysiologic feature of CSAs is the iron-driven increase of ROS in mitochondria, leading to cell damage and impaired erythropoiesis. Although deposition of non-heme iron in the mitochondria of erythroid precursors is present in all cases of CSA, systemic iron overload does not always develop in untransfused patients but occurs mainly when the inherited defect is restricted to the erythroid cell line (*XLSA*, *GLRX5*, and *SLC25A38* mutations), as a consequence of inefficient erythropoiesis and increased iron absorption [3]. The main site of iron accumulation is the liver, which in untreated patients is at risk of chronic damage—progressing to fibrosis and cirrhosis—whereas cardiac iron overload is much rarer [170].

### 4.1. ALAS2-Related X-Linked Sideroblastic Anaemia

First described by Cooley in 1945 [174], the X-linked sideroblastic anemia (XLSA) related to pathogenic variants in the erythroid-specific form of aminolevulinate synthase (*ALAS2*) is the most common form of CSA [169]. The prevalence of XLSA is unknown: about 200 cases and fewer than 100 unrelated probands have been described in the literature [166].

In the last few decades, more than 100 different pathogenic variants have been reported, most of which are missense, loss-of-function mutations affecting the ALAS2 catalytic domain, the interaction site between ALAS2 and succinyl-CoA synthetase, or the binding domain for pyridoxal phosphate [169,175]. The presence of the latter mutation site accounts for pyridoxin-responsiveness in about two-thirds of patients with XLSA [176]. Additionally, pathogenic variants in the *ALAS2* promoter have been reported in about 5% of congenital sideroblastic anemias [177]: recently, two variants in a new enhancer region containing a GATA binding site have been identified as responsible for a decrease in *ALAS2* transcription and sideroblastic anemia in male patients [178].

Due to an X-linked pattern of inheritance, XLSA presents mainly in hemizygous males, during childhood or adolescence, with a microcytic, hypochromic anaemia and signs of iron overload [3].

Female carriers of *ALAS2* mutations are usually asymptomatic, but occasionally they may have a late presentation of CSA as a result of familial-skewed inactivation of the normal X-chromosome, also referred to as “unfortunate skewing” [167]. The phenotype of women heterozygous for an *ALAS2* mutation depend on the percentage of cells in which the normally functioning X chromosome is inactivated, as well as on the impact of the respective mutation. Elderly-onset XLSA has also been described—in fact, age has been identified as an additional cause of acquired skewed lyonization in hematopoietic cells [179]. Interestingly, most females with XLSA exhibit a macrocytic phenotype, possibly related to an accelerated, EPO-driven release of premature erythrocytes from the bone marrow in conditions of anemic hypoxia. The severity of the mutations leading to XLSA in females is responsible for the complete absence of mutant erythrocytes in peripheral blood. Moreover, these mutations would be lethal in affected males, which is why no males with XLSA are present in the families of female probands [180].

As previously anticipated, in about two-thirds of XLSA cases, pyridoxine (vitamin B6) supplementation induces a gradual increase in hemoglobin levels up to normalization. Severe anemia, unresponsive to pyridoxine, requires chronic transfusion therapy, which inevitably leads to secondary iron overload and a need for iron chelation therapy [176].

#### 4.1.1. X-Linked Sideroblastic Anaemia with Ataxia

A rare form of XLSA has been associated with pathogenic variants of *ABCB7*: cerebellar ataxia, incoordination, and diminished deep tendon reflex may be evident by the first year of age but are not progressive. The degree of anemia is milder than in *ALAS2*-related XLSA and is associated with elevated levels of erythrocyte protoporphyrin [170].

Recently, ABCB7 has been identified as an actor in the mitochondrial complex formation (see Section 2.2): a connection with the carboxy-terminal region of ferrochelatase has been demonstrated, leading to speculation about a regulating function in the export of [Fe-S] clusters from the mitochondrial to the cytoplasmic space. Iron, trapped in mitochondria, is responsible for the peculiar erythrocyte modifications; the increase in erythrocyte protoporphyrin levels would suggest a normal ALAS2 activity associated with some degree of iron unavailability for FECH, i.e., for its [Fe-S] clusters [181].

XLSA with ataxia (XLSA/A) is not associated with systemic iron overload: in general, anemia is a secondary component in syndromic forms of CSA, and iron overload is usually not reported [3].

### 4.2. Autosomal Recessive Sideroblastic Anaemias

#### 4.2.1. *SLC25A38* Mutations

Pathogenic variants of the erythroid-specific mitochondrial transporter *SLC25A38*, located on chromosome 3 (3p 22.1), have been reported in a group of patients with severe microcytic anemia and systemic iron overload in early childhood [169].

SLC25A38 is a mitochondrial glycine transporter, preferentially expressed in transferrin receptor (CD71) positive cells: its absence has been associated with decreased ALA levels, despite normal ALAS2 enzymatic activity, decreased heme production, consistent with the essential role of glycine as a substrate in the first step of heme synthesis (see Section 2.2).

A recent case series reported that CSA associated with *SLC25A38* mutations usually presents at birth or infancy (<12 months) with severe reticulocytopenic anemia requiring chronic transfusion. Iron overload developed in all patients surviving childhood and required chelation therapy. Allogenic hematopoietic stem cell transplantation has been successfully performed in some of the most severe patients [182].

Guernsey et al. first described loss-of-function mutations in *the SLC25A38* gene, causing a non-syndromic autosomal recessive form of CSA in 12 patients with unexplained CSA. They previously demonstrated, with mRNA knockdown experiments in zebrafish embryos, that simultaneous injection of morpholinos directed to the two zebrafish genes *scl25a38* orthologs lead to an anemic phenotype [183]. Furthermore, the authors reported that yeast deletion strain could not reduce sodium nitroprusside (in a heme-dependent reaction used as a surrogate of heme biosynthesis) unless supplemented with glycine or ALA [183,184].

#### 4.2.2. *GLRX5*, *HSPA9*, *HSCB* Mutations

[Fe-S] clusters are an essential component in many mitochondrial and extra-mitochondrial processes (see Section 2.2). Alterations in this complex have been associated with neurologic and metabolic impairment, e.g., in Friedreich ataxia [55] and mitochondrial myopathies [185].

Other than *ABCB7* mutations causing XLSA/A (see Section 4.2), pathogenic variants in three additional proteins involved in the transfer of [Fe-S] clusters have been associated with non-syndromic congenital sideroblastic anemia.

So far, only three cases of CSA caused by mutations in glutaredoxin-5 (GLRX5) have been described [186,187,188]: two men that, at the age of 29 and 44 years, respectively, presented with severe microcytic hypochromic anemia and clinical signs of iron overload (bronze diabetes, hypogonadism, hepatosplenomegaly, and cirrhosis), along with a 14-year old girl with anemia, elevated serum ferritin level and hepatic iron overload (LIC 200 micromol/g), without clinical signs of organ damage. In all three cases, a significant clinical improvement has been reported after chelation therapy with deferoxamine (DFO) was started: serum ferritin level and liver iron content gradually decreased, whereas hemoglobin levels increased until transfusions and iron chelation were postponed or stopped.

Glutaredoxins (GRX) are small proteins with thiol reductase activity involved in the reduction of oxidized glutathione and other disulfide bonds; however, their function in [Fe-S] cluster biogenesis has not been completely assessed yet. Ye and colleagues proposed that GLRX5 could act as a scaffold, delivering [2Fe-2S] cluster to different mitochondrial proteins, mainly FECH [189].

*GLRX5* knockdown experiments, conducted in HeLa S3 cells, revealed a significant decrease in mitochondrial aconitase enzymatic activity without reduction of aconitase levels. A relevant increase in total non-heme iron was observed and related to mitochondrial iron overload, whereas cytosolic iron levels were normal. In line with the cytosolic iron-depleted state and [Fe-S] cluster deficiency, a marked increase in IRP levels was observed, associated with ALAS2 and ferritin repression and TFR1 RNA stabilization [189].

In erythroid K562 cells, GLRX5 RNA-interference induced a decreased expression of ALAS2, FECH, and ferritin: this observation supported the specific role of GLRX5 in the heme biosynthetic pathway in hematopoietic cells.

Interesting data emerged from gene profiling in GLRX5-deficient K562 cells: ferroportin transcript levels were markedly increased, likely in response to stress caused by the alteration in mitochondrial iron homeostasis. The same transcriptional upregulation was not observed in HeLa S3 cells and has been attributed to a newly identified FPN1 mRNA variant, *FPN1b*, which does not contain an IRE and therefore evades IRP-mediated repression [189]. The specificity of FPN1b and ALAS2 for erythroid cells may account for the erythroid-specific phenotype of GLRX5 mutations.

In conclusion, GLRX5 mutations impair the assembly of [Fe-S] clusters and are associated with reduced ferrochelatase and mitochondrial aconitase activities. Reduction in heme biosynthesis and mitochondrial iron overload, with sideroblast formation, may be considered as expected consequences; however, total porphyrin and PPIX levels are maintained unexpectedly low despite the significant reduction of FECH activity, probably because of a reduction in ALAS2 activity [188].

Loss-of-function mutations of HSPA9 inherited as an autosomal recessive trait and, in some cases, with a pseudodominant pattern, have been described in 11 families with CSA [190]. A single patient with CSA and mild pancytopenia due to a frameshift variant on the heat-shock cognate B (HSCB) promoter has been described [191]. HSPA9 is a heat-shock protein family member (also known as mortalin), which seems to act as a chaperone for [2Fe-2S] clusters from the mitochondrial scaffold proteins to GLRX5. HSBC, instead, functions as a co-chaperone, stimulating the ATPase activity of HSPA9 and facilitating the release of [Fe-S] clusters [61,166].

In shiraz zebrafish mutants for *glrx5*, the overexpression of ALAS2 RNA without the IRE in the 5${}^{\prime }$-UTR rescued the production of hemoglobin [192]; the same benefit has been observed in *hscb* knockdown experiments in zebrafish embryos [61]. It may be speculated whether targeting the IRE in ALAS2, as well as limiting IRP-mediated repression on the translation of proteins which are crucial to heme biosynthesis and iron regulation, could be some successful strategies to prevent anemia in CSA due to mitochondrial [Fe-S] cluster defects.

#### 4.2.3. *STEAP3*-Related Sideroblastic Anaemia with Primary Hypogonadism and DMT1 Deficiency

To complete this review, an outline is provided of two rare forms of iron-loading microcytic anaemia associated with defects in the TF-TFR1 endosomal cycle (only *STEAP3*-related anemia is associated with ringed sideroblasts) [193].

A defect in *STEAP3*, which encodes a ferroreductase involved in iron uptake in erythroblasts, has been associated with a severe form of hypochromic microcytic anemia with ringed sideroblasts. In 2005, Ohgami et al. demonstrated that mouse mutant *nm1054* was associated with a severe form of hypochromic microcytic anemia caused by an impairment in ferroreductase activity, which could be corrected by the induction of *Steap3* overexpression [194]. A few years later, the first and so far only human *STEAP3* mutation has been described in three siblings, two males and one female, presenting since infancy or adolescence with a severe form of transfusion-dependent hypochromic microcytic anemia, with iron overload and primary hypogonadism. Blood marrow smears showed some ringed sideroblasts, although the mechanism leading to mitochondrial iron deposition remains unexplained. Since both parents were healthy, a recessive pattern of transmission has been suggested. In fact, a heterozygous nonsense (p.Cys100Stop) mutation in *STEAP3* was found in the probands as well as their father, whereas only the probands displayed low levels of STEAP3 mRNA in the blood. Instead, no clearly pathogenic variant was identified in the mother, so the authors suggested that the coinheritance of a paternal mutated allele and a maternal hypomorphic or weakly expressed allele could be responsible for the clinical manifestations in the probands [195]. All the siblings required regular blood transfusions, EPO administrations to lengthen the transfusion intervals and chronic iron chelation therapy [195].

Ten patients suffering from hypochromic microcytic anemia, sometimes already present at birth, have been described as harboring mutations in *SLC11A2*. All individuals but two presented with liver iron overload (in the two more recent cases, liver iron levels have not been assessed yet) [196]. *SLC11A2* encodes for divalent metal transporter 1 (DMT1), a ferrous iron importer in erythroblasts, duodenal cells, and macrophages. It was first identified in the *mk* mouse model, in which a missense mutation of the transmembrane domain of the protein was associated with severe iron-deficient anemia [197]. To date, at least four patients with DMT1 deficiency have been treated with EPO administration, obtaining a significant amelioration of anemia and stopping blood transfusions in a few of them [196]. However, a comparable decrease in liver iron overload has not been obtained as iron chelation therapy has been ineffective or led to a sharp decrease in hemoglobin levels [193,198,199].

## 5. Conclusions

From a biochemical standpoint, proper handling of iron is essential to the health of eukaryotes. To perform this task effectively, heme has been perfected by evolution as a versatile iron-containing coordination complex and an irreplaceable prosthetic group for countless reactions which rely on the properties of iron. Understandably, heme and iron reciprocally regulate each other’s metabolism through sophisticated molecular machinery which intervenes at all levels of both biochemical pathways.

In conditions of altered heme biosynthesis, iron plays a pivotal role in several mechanisms of damage. Alterations in iron metabolism are a feature of several kinds of porphyrias as well as congenital sideroblastic anemias, and pharmacological alterations of iron metabolism can have wide-ranging clinical consequences, either in positive (as in CEP), negative (as in hemin treatment in AHPs), or still undefined directions.

Deepening our understanding of the interplay between iron and heme metabolism can help to shed light not only on some as-yet-unknown details of their biochemical roles in living beings but also on the pathogenic mechanisms of the disorders of heme biosynthesis in humans and ultimately pave the way to finding more effective therapeutics for the treatment of this heterogeneous group of diseases. 

## Abbreviations

5${}^{\prime }$-UTR	5${}^{\prime }$ untranslated region
ABCB	adenosine triphosphate-binding cassette subfamily B
ACTR	activin receptor
AIP	acute intermittent porphyria
AHPs	acute hepatic porphyrias
ALA	δ-aminolevulinic acid
ALA·	enoyl (ALA) radical
ALAD	ALA dehydratase
ALAS1	ALA synthase 1
ALAS2	ALA synthase 2
ALK	activin receptor-like kinase
APA	acute porphyric attack
ARCSA	autosomal recessive sideroblastic anaemia
BMP	bone morphogenetic protein
CEP	congenital erythropoietic porphyria
ClpX	caseinolytic mitochondrial matrix peptidase chaperone subunit X
CP	ceruloplasmin
CPOX	coproporphyrinogen III oxidase
CSA	congenital sideroblastic anaemias
CYP1A2	cytochrome 1A2 of the P450 family
DCYTB	duodenal cytochrome B
DFO	deferoxamine
DMT1	divalent metal transporter 1
DOVA	4,5-dioxovaleric acid
EPP	erythropoietic protoporphyria
EPO	erythropoietin
ERFE	erythroferrone
Fe	iron
FECH	ferrochelatase
Fe-S cluster	iron-sulfur cluster
F-PCT	familial porphyria cutanea tarda
FPN1	ferroportin (protein)
FTH1	ferritin heavy chain
FTL	ferritin light chain
FXN	frataxin
GATA1	GATA-binding factor 1
GLRX5	glutaredoxin 5
GDF15	growth/differentation factor 15
GRX	glutaredoxin
HAMP	hepcidin gene
HCP	hereditary coproporphyria
HC	hemochromatosis
HEPH	hephaestin
HJV or HFE2	hemojuvelin gene
HFE	human homeostatic iron regulator protein
HMB	hydroxymethylbilane
HMBS	HMB synthase
HO	heme oxygenase
HSCB	heat-shock cognate B
HSPA9	heat-shock protein family A member 9
IL-6	interleukin 6
IRE	iron responsive element
IRIDA	iron deficiency-iron refractory anaemia
IRP1	IRE-binding protein 1
IRP2	IRE-binding protein 2
LIC	liver iron content
LONP1	lon peptidase 1, mitochondrial
LSEC	liver sinusoidal endothelial cell
Mfrn1	mitoferrin 1
MtF	mitochondrial ferritin
NCOA4	nuclear receptor coactivator 4
PBG	porphobilinogen
PCBP1	poly rC–binding protein 1
PCT	porphyria cutanea tarda
PLP	pyridoxal phosphate
PPOX	protoporphyrinogen oxidase
RISC	RNA-induced silencing complex
ROS	reactive oxygen species
S-PCT	sporadic porphyria cutanea tarda
SLC40A1	ferroportin (gene)
SMAD	suppressor of mothers against decapentaplegic
STAT3	signal transducer and activator of transcription 3
STEAP3	six-transmembrane epithelial antigen of prostate 3
TF	transferrin
TFR1	transferrin receptor 1 (protein)
TFR2	transferrin receptor 2
TFRC	transferrin receptor (gene)
TMPRSS6	transmembrane protease serine 6
UROD	uroporphyrinogen decarboxylase
UROS	uroporphyrinogen III synthase
UTR	untranslated region
VP	variegate porphyria
XLP	X-linked protoporphyria
XLSA	X-linked sideroblastic anaemia
XLSA/A	XLSA with ataxia
